# The ESX-4 substrates, EsxU and EsxT, modulate *Mycobacterium abscessus* fitness

**DOI:** 10.1371/journal.ppat.1010771

**Published:** 2022-08-12

**Authors:** Marion Lagune, Vincent Le Moigne, Matt D. Johansen, Flor Vásquez Sotomayor, Wassim Daher, Cécile Petit, Gina Cosentino, Laura Paulowski, Thomas Gutsmann, Matthias Wilmanns, Florian P. Maurer, Jean-Louis Herrmann, Fabienne Girard-Misguich, Laurent Kremer

**Affiliations:** 1 Université Paris-Saclay, UVSQ, Inserm, Infection et inflammation, Montigny-Le-Bretonneux, France; 2 Centre National de la Recherche Scientifique UMR 9004, Institut de Recherche en Infectiologie de Montpellier (IRIM), Université de Montpellier, Montpellier, France; 3 National and WHO Supranational Reference Center for Mycobacteria, Research Center Borstel, Leibniz Lung Center, Borstel, Germany; 4 INSERM, IRIM, Montpellier, France; 5 European Molecular Biology Laboratory, Hamburg Unit, Hamburg, Germany; 6 Research Center Borstel, Leibniz Lung Center, Division of Biophysics, Borstel, Germany; 7 Institute of Medical Microbiology, Virology and Hospital Hygiene, University Medical Center Hamburg-Eppendorf, Hamburg, Germany; 8 APHP, GHU Paris-Saclay, Hôpital Raymond Poincaré, Service de Microbiologie, Garches, France; University of Washington, UNITED STATES

## Abstract

ESX type VII secretion systems are complex secretion machineries spanning across the mycobacterial membrane and play an important role in pathogenicity, nutrient uptake and conjugation. We previously reported the role of ESX-4 in modulating *Mycobacterium abscessus* intracellular survival. The loss of EccB4 was associated with limited secretion of two effector proteins belonging to the WXG-100 family, EsxU and EsxT, and encoded by the *esx-4* locus. This prompted us to investigate the function of *M*. *abscessus* EsxU and EsxT *in vitro* and *in vivo*. Herein, we show that EsxU and EsxT are substrates of ESX-4 and form a stable 1:1 heterodimer that permeabilizes artificial membranes. While expression of *esxU* and *esxT* was up-regulated in *M*. *abscessus*-infected macrophages, their absence in an *esxUT* deletion mutant prevented phagosomal membrane disruption while maintaining *M*. *abscessus* in an unacidified phagosome. Unexpectedly, the *esxUT* deletion was associated with a hyper-virulent phenotype, characterised by increased bacterial loads and mortality in mouse and zebrafish infection models. Collectively, these results demonstrate that the presence of EsxU and EsxT dampens survival and persistence of *M*. *abscessus* during infection.

## Author summary

The evolution of bacterial secretion systems is strongly associated with the intracellular lifestyle and interactions of bacteria with their hosts. Here, we show that an ancestral type VII secretion system, ESX-4, modulates the pathophysiology of *Mycobacterium abscessus* infection, which is directly related to its major effectors, EsxU and EsxT. While EsxU/EsxT were found to permeabilize lipid bilayers, deletion of the *esxUT* genes increased the virulence of *M*. *abscessus* in animal models. In support of these observations, strictly pathogenic mycobacteria in humans and animals, such as *Mycobacterium tuberculosis* or *Mycobacterium marinum* have evolved without evidence of secretion of their respective EsxU/EsxT substrates. Together, these results underscore the possible *in vivo* advantage procured by the absence of EsxU and EsxT in strictly pathogenic mycobacterial species.

## Introduction

Type VII secretion systems (T7SS), also known as Early Antigenic Secretion (ESX) systems, are well-conserved among mycobacterial species and are known to be essential for protein secretion. To date, five ESX systems have been reported in mycobacteria, namely ESX-1 to ESX-5. T7SS are structurally composed of at least four ESX-conserved structural components (EccB, EccC, EccD, EccE), as recently demonstrated for ESX-3 and ESX-5 [1–3], and further stabilized by a conserved membrane-bound mycosin protease (MycP) [4], ESX-type-specific associated proteins (Esp), and secreted/exported effectors organized in an operonic arrangement. However, not all mycobacteria possess the five *esx* loci. Strictly pathogenic species have four ESX systems (*Mycobacterium marinum esx-1-3-4-5*) or five ESX systems (*Mycobacterium tuberculosis* complex (*esx-1-2-3-4-5*) [5,6] whereas opportunistic pathogens such as *Mycobacterium abscessus* possess only two (*esx-3-4*) [7]. In addition, some loci, such as *esx-1*, involved in the virulence of strict pathogens, are also present in saprophytic species, such as *Mycobacterium smegmatis*. Different roles have been ascribed to ESX sytems, individually or in association with each other, such as virulence, nutrient and ion uptake, toxin trafficking and secretion and mycobacterial conjugation [8–14]. One of the most studied examples is ESX-1 from *M*. *tuberculosis*. The *esx-1* operon contains two antigenic effector proteins, EsxA and EsxB, previously named the 6-kDa Early Secretory Antigenic Target (ESAT-6) and 10-kDa Culture Filtrate Protein (CFP-10), respectively. EsxA and EsxB form a 1:1 heterodimeric complex with phagosomal membrane-permeabilization activity, which contributes to the phagosomal escape of pathogenic mycobacteria [8,15,16].

Phylogenetic analyses suggest that *esx-4* is the ancestral locus from which all other mycobacterial ESX systems have evolved [6]. Until recently, ESX-4 was considered as non-functional in pathogenic mycobacteria, such as *M*. *tuberculosis* [8]. Comparatively, deletion of one of the structural components of the *M*. *abscessus* T7SS ESX-4, EccB4, resulted in decreased intracellular survival, with impaired phagosomal membrane rupture and phagosomal acidification [17]. However, the underlying molecular mechanism explaining the *ex vivo* attenuated phenotype of the Δ*eccB4* mutant remains to be established. Noteworthingly, Δ*eccB4* was associated with reduced secretion of two *esx4*-encoded proteins, EsxU and EsxT, in the culture supernatant, suggesting a link between decreased intracellular replication of Δ*eccB4* and the level of EsxU/EsxT secretion [17].

*M*. *abscessus* represents an excellent surrogate to examine the role of ESX-4 effectors with respect to host-pathogen interactions and persistence in the host, involvement in membrane interaction and phagosomal escape and virulence. Critically, deletion of *esxUT* resulted in enhanced virulence in infected mice and zebrafish, characterised by increased bacterial burdens and mortality. These findings open up the attractive hypothesis that the inability to secrete these two ESX-4 substrates may have led to the emergence of increased virulence in strictly pathogenic mycobacteria.

## Results

### *M*. *abscessus* EsxU and EsxT co-expression and secretion

**Fig 1:**
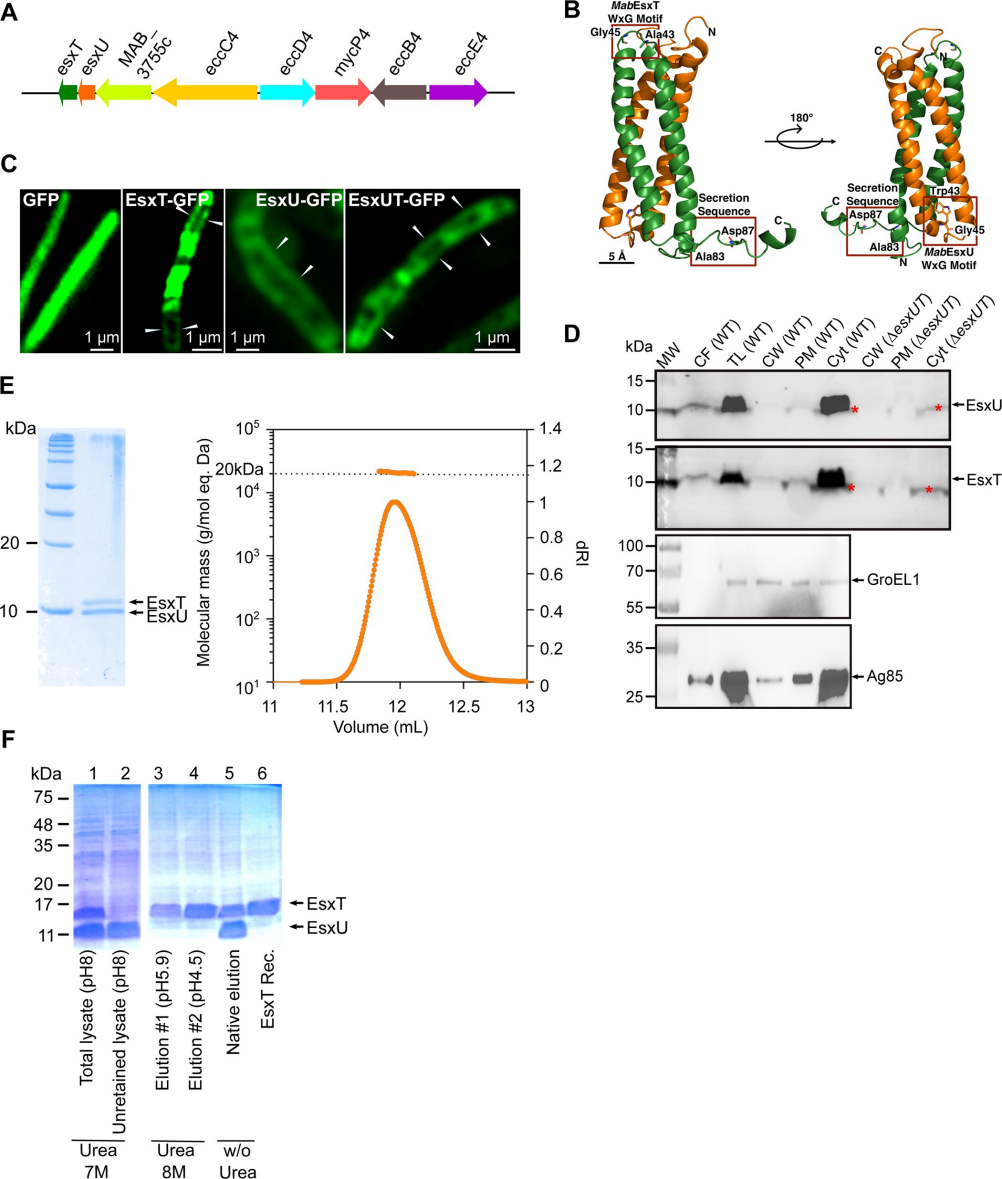
*M*. *abscessus* EsxU and EsxT are co-expressed and secreted by ESX-4. (A) Schematic representation of the *M*. *abscessus esx-4* gene cluster. (B) Model of *M*. *abscessus* EsxU/EsxT made using ProMod version 3 3.2.10 and based on the NMR structure of *M*. *tuberculosis* EsxAB (PDB code: 1wa8.1). EsxU and EsxT are shown in orange and green, respectively, and the essential residues of the WXG motifs and the secretion sequence are represented in boxes. (C) Representative confocal microscopy images of Δ*esxUT* strains expressing either GFP, EsxT-GFP, EsxU-GFP or EsxUT-GFP to illustrate protein localization. A series of Z-stacks were collected with a Zeiss LSM880 Airyscan confocal microscope and the images were assembled and reconstructed with Zen blue software. (D) Immunoblotting showing the localization of EsxU and EsxT in different fractions: culture filtrate (CF), total lysate (TL), cell wall (CW), plasma membrane (PM) and cytosol (Cyt). Western blotting of subcellular fractions from the Δ*esxUT* strain show the lack of EsxU and EsxT subunits. Asterisks indicate non-specific bands. The cytosolic marker GroEL1 and the secreted protein Antigen 85B were included as controls. MW, molecular weight marker. (E, left) Co-purified *M*. *abscessus* EsxU/EsxT showing bands at approximately 10 kDa in 15% Coomassie-stained SDS-PAGE. Both genes were cloned and expressed in *M*. *smegmatis*. (Right) EsxU/EsxT heterodimerization were observed by SEC-MALLS. (F) Ni-NTA column purification of His-tagged EsxT alone in denaturing condition (with urea) and co-purification of EsxU and EsxT in native condition (without urea) in *E*. *coli*.

EsxU and EsxT are encoded by the *M*. *abscessus esx-4* locus [5] (Fig 1A) and belong to the WXG-100 family, similarly to the *M*. *tuberculosis* EsxA and EsxB effectors. These proteins are characterised by the presence of the characteristic WXG (tryptophan-X-glycine) motif in EsxU and a secretory signal motif (HxxxD/ExxhxxxH) in the C-terminus of EsxT [18] (Fig 1B). Previous work indicated that the EsxU and EsxT protein levels were slighty reduced in the secretome of *M*. *abscessus* Δ*eccB4*, suggesting that they are, at least partially, secreted through the ESX-4 machinery [17].

Initially, we sought to visualise the localization of EsxU and EsxT on the surface of *M*. *abscessus* using the Δ*esxUT* mutant, which was then individually complemented with GFP-tagged EsxT or EsxU (S1A Fig). Probing the crude lysates from the various strains with anti-GFP antibodies revealed a band of the expected size, corresponding to EsxU-GFP or EsxT-GFP proteins (S1B Fig). Using confocal microscopy, we observed dispersed green fluorescent areas for EsxU-GFP, EsxT-GFP, or EsxUT-GFP as well as exclusively detectable cell wall labeling (Fig 1C, white arrows). This contrasts with the green fluorescent signal of GFP alone, which was homogenously distributed along the longitudinal axis of the bacilli (Fig 1C). The intensity of the EsxU-GFP signal was strongly reduced compared with the EsxT-GFP signal, in agreement with the Western blot analysis using anti-GFP antibodies (S1B Fig).

Proteins resulting from subcellular fractionations or concentrated culture filtrates were then probed with specific mouse polyclonal antibodies to reveal the presence of EsxU and EsxT. EsxU- and EsxT-specific bands were mainly found in the culture filtrate and total lysate (Fig 1D). The low accumulation of EsxU and EsxT subunits in cell wall and plasmic membrane fractions suggests that, unlike their native promoter, the *hsp60* promoter, which allows constitutive expression of GFP-tagged versions, may artificially increase their respective association at the bacilli’s periphery (Fig 1C).

As described for other low molecular weight Esx substrates [19,20], EsxU and EsxT were co-purified using *M*. *smegmatis* as a heterologous expression system (Fig 1E, left panel). The proteins were identified by mass spectrometry and subsequently analysed by Multiple angle laser light scattering (SEC-MALLS), which showed the presence of an approximately 20-kDa heterodimer in solution (Fig 1E, right panel). EsxU and EsxT from *M*. *abscessus* were also co-expressed in *Escherichia coli* where EsxT was co-purified with EsxU under native conditions or alone under denaturing conditions after elution at pH 5.9 and 4.5, whereas EsxU was found in the unretained column lysate (Fig 1F). The strong association between EsxU and EsxT was further confirmed by GFP-pull-down experiments performed in *M*. *abscessus* Δ*esxUT* complemented with pVV16-*esxUT-GFP* and followed by mass spectrometry analysis, with EsxT being physically associated with EsxU (S1C–S1E Fig). This analysis suggests that EsxT may also act in concert with additional protein partners (S1E Fig). Collectively, these data indicate that an EsxU/EsxT heterodimer is expressed in *M*. *abscessus* and is translocated across the plasma membrane and secreted into the culture filtrate.

### EsxU and EsxT are involved in phagosomal membrane damage in macrophages

**Fig 2:**
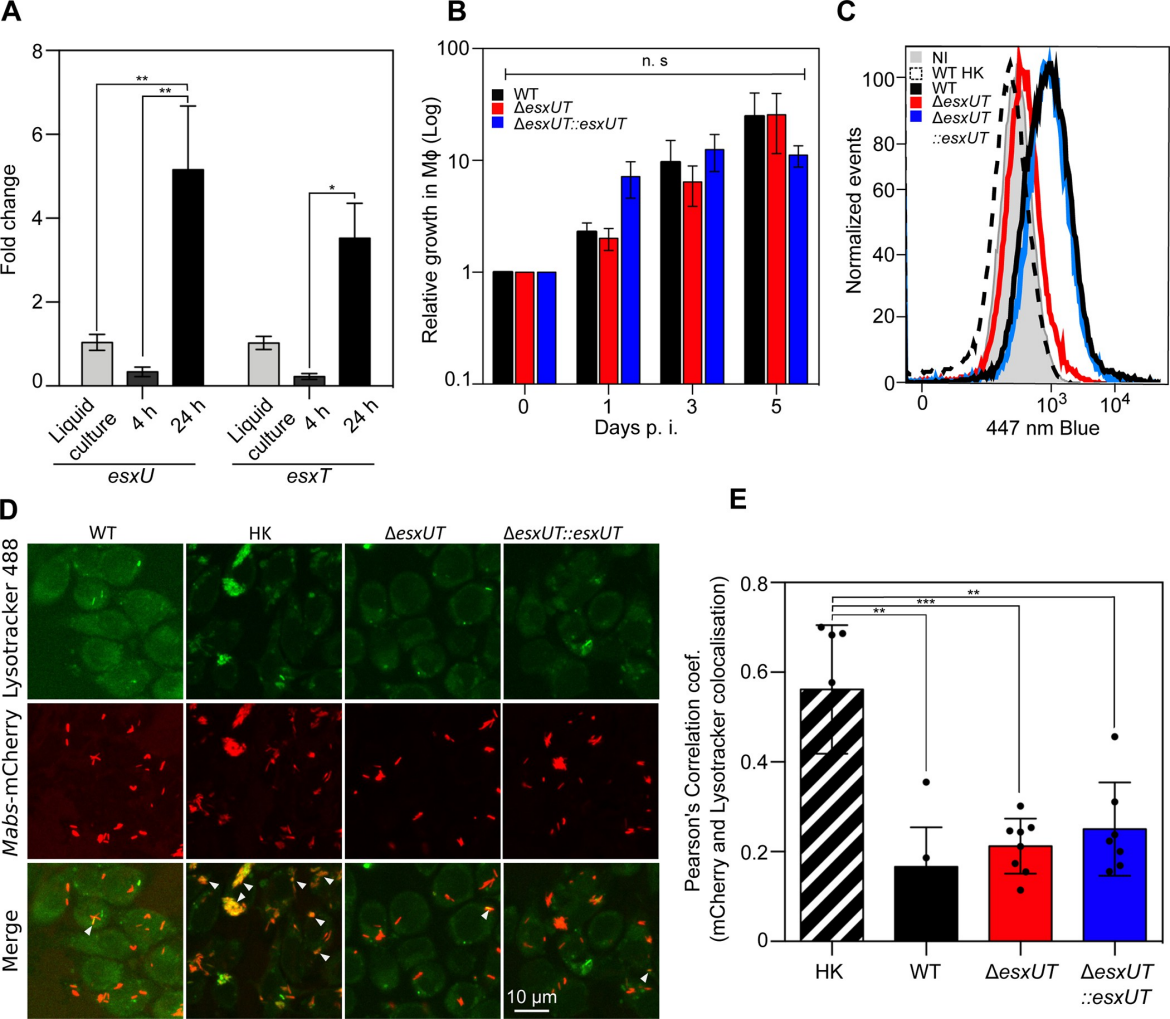
Δ*esxUT* is maintained in an intact phagosome without acidification in macrophages. (A) Expression of *esxU* and *esxT* genes in J774.2 macrophages at 4 hrs and 24 hrs post-infection (black) relative to planktonic growth expression in broth medium (gray). (B) Relative intracellular survival of WT (black), Δ*esxUT* (red), and Δ*esxUT*::*esxUT* (blue) strains as determined by CFU counts during infection in J774.2 macrophages at an MOI of 10:1. (C) Phagosomal rupture detected by CCF-4 FRET-based flow cytometry 24 hrs post-infection. Results are depicted as signal overlays per group with 1,000,000 events per condition acquired in WT (black line), heat-killed (dotted line), Δ*esxUT* (red line), Δ*esxUT*::*esxUT* (blue line) strains and in uninfected cells (NI, gray line). (D) Colocalization of *M*. *abscessus* WT, heat-killed, Δ*esxUT* and Δ*esxUT*::*esxUT* strain expressing mCherry with the acidotropic dye LysoTracker Green in infected J774.2 cells 3 hrs post-infection. Arrows indicate intracellular mycobacteria co-localizing with LysoTracker green. (Scale bar, 10 μm). (E) mCherry-labeled strains colocalized with LysoTracker were measured by Pearson correlation of at least 100 infected cells in 10 different fields. Data are representative of three (B and C) or two (D and E) independent experiments and represent means ± SEM. *P* values were determined by ANOVA with Tukey’s test using GraphPad prism program (A and B) and unpaired t test (E); ns, not significant; *P < 0.05, **P < 0.01, ***P < 0.001, ****P < 0.0001.

In *M*. *tuberculosis*, the ESX-1 substrates, EsxA and EsxB, promote intracellular survival and phagosomal escape [21], which prompted us to investigate the functions of EsxU and EsxT in macrophages. To determine the expression levels of *esxU* and *esxT* during macrophage infection, qRT-PCR analysis was performed after infection of macrophages, which showed upregulation of both transcripts at 24 hours post-infection (hpi) (Fig 2A). While these results indicate that *esxU* and *esxT* are transcribed under intracellular conditions, it may be speculated that the EsxU/EsxT heterodimer plays a role in intracellular replication of *M*. *abscessus*. To test this hypothesis, a *esxUT* deletion mutant (Δ*esxUT*) was generated (S2A and S3A and S3B Figs) along with the corresponding complemented strains (Δ*esxUT*::*esxUT*) (S3C and S3D Fig). As anticipated, this mutant failed to produce EsxU and EsxT (Fig 1D). Macrophages were then infected with the wild-type (WT), Δ*esxUT*, or Δ*esxUT*::*esxUT* strains (Fig 2B). Δ*esxUT* showed no morphotypic changes on plates or growth defects in broth medium compared with the WT strain (S2B and S2C Fig). Furthermore, there were no major differences in the intracellular growth between the three strains (Figs 2B and S3E). Similar results were obtained in amoebae, a unicellular phagocytic cell type regarded as a potential environmental reservoir, possibly involved in shaping the *M*. *abscessus* virulence profile for intracellular adaptation [17,22,23] (S2D Fig).

Next, we examined the potential effect of EsxU/EsxT on phagosomal membrane disruption using a FRET-based assay [17]. As shown in Figs 2C and S3F, Δ*esxUT* and heat-killed WT *M*. *abscessus* were unable to damage the phagosomal membrane, unlike the WT or Δ*esx-UT*::*esxUT* strains. However, Δ*esxUT* remained present in a non-acidified compartment, as evidenced by confocal microscopy, similar to the WT strain, but to a lesser extent than heat-killed *M*. *abscessus* (Fig 2D and 2E). Altogether, these results suggest that the loss of EsxU and EsxT abolishes phagosomal membrane damage but maintains the blockage of phagosomal acidification.

### EsxU/EsxT interacts with artificial eukaryotic membranes

**Fig 3:**
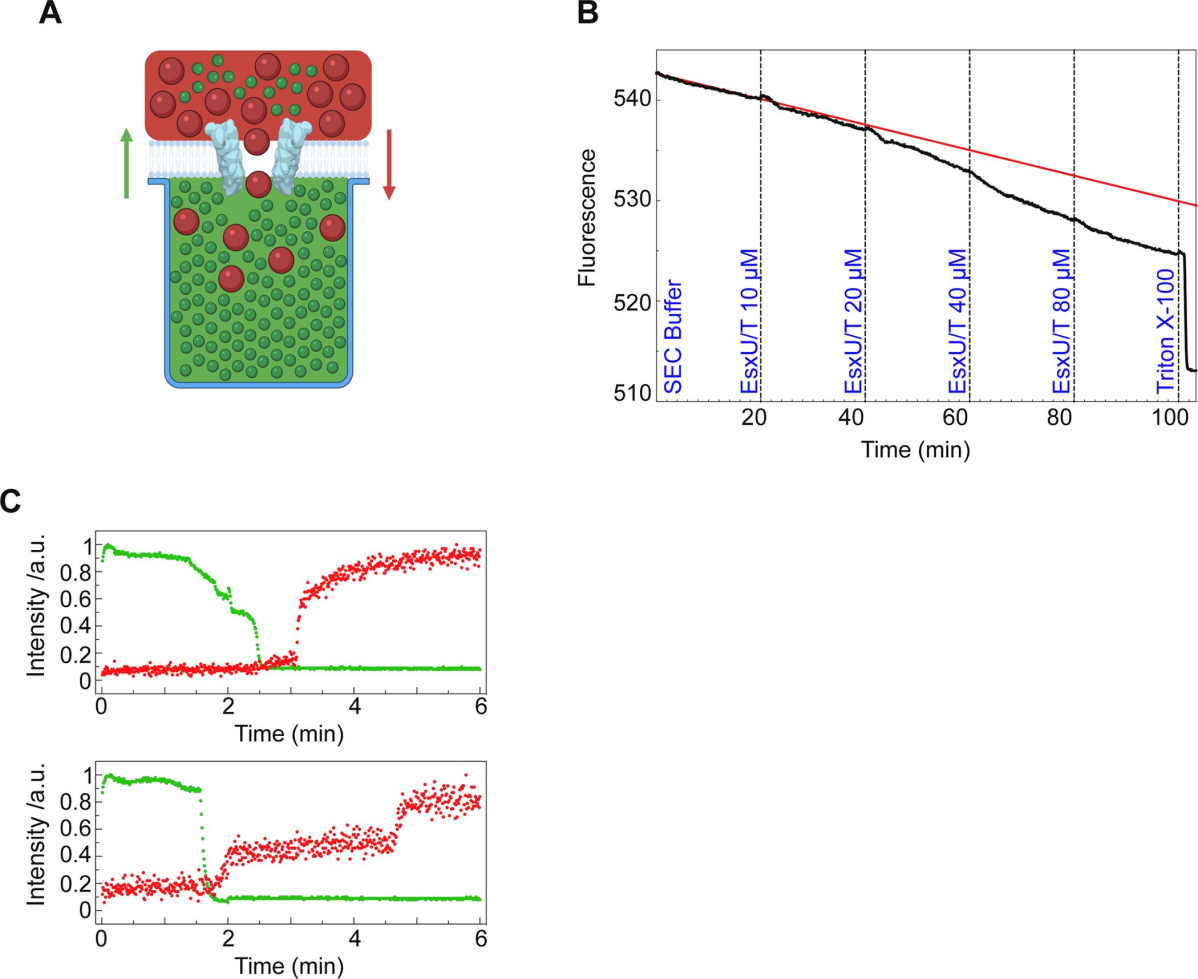
Interaction of the *M*. *abscessus* EsxU/EsxT complex with DOPC bilayers. (A) Schematic of the NanoSpot method. DOPC bilayers were built over cavities filled with a fluorescent dye (green). A second fluorescent dye of known size (red) was added to the top of the membrane prior to addition of the protein complex. Fluorescent signals were continuously measured. (B) Dye influx and efflux using the NanoSpot technique. DOPC bilayers were formed at 37°C and pH 7.2 on top of cavities filled with Atto488 carboxy fluorescence dye. Fluorescence (black curve) decreased as more protein was added (final EsxU/EsxT concentration is indicated in blue). Upon addition of Triton X-100, fluorescence decreased abruptly. The red curve represents the trend line of fluorescence loss by photo-bleaching if no protein had been added. Over 300 cavities covered with DOPC membrane are plotted in this graph. (C) 40, 000 MW TexasRed-labelled dextran was added to the media outside the cavities before the EsxU/EsxT complex was supplemented to the well (final concentration: 10 μM). Green dots represent efflux of Atto488 carboxy efflux dye and red dots represent influx of TexasRed dye. Each graph represents one cavity.

To decipher the properties of the EsxU/EsxT heterodimer and its potential interaction with biological membranes, biophysical experiments were performed using dioleoyl-phosphatidylcholine (DOPC) as a model of eukaryotic membranes [24] as well as the EsxU/EsxT complex expressed and purified from *M*. *smegmatis*. The heterodimer-membrane interaction was then studied using the NanoSpot approach (Fig 3A). The intensity of a fluorescent dye trapped in cavities underneath a DOPC bilayer was monitored. We observed a decrease in fluorescence upon addition of purified EsxU/EsxT, suggesting a direct interaction with the membrane that allowed the dye to escape (Fig 3B). The decrease in intensity by photo-bleaching of the fluorescence dye is indicated by the red curve. To verify whether the complex formed an organized structure, such as a pore, and did not just perforate the membrane in a single event by complete membrane destruction, a second fluorescent dye (larger dextran conjugate) was added above the membrane. Single-cavity analysis showed an increase in fluorescence of the second dye in several cavities (two cavities represented), indicating dextran influx. The concentration dependence is not linear, demonstrating that the pore formation depends on a critical local aggregation of the proteins on/in the membrane [24]. Taken together, this demonstrates that EsxU/EsxT complexes are capable of forming stable higher-order oligomeric structures in lipid bilayers (Fig 3C).

### Deletion of *esxUT* leads to a hypervirulent phenotype in different animal models

**Fig 4:**
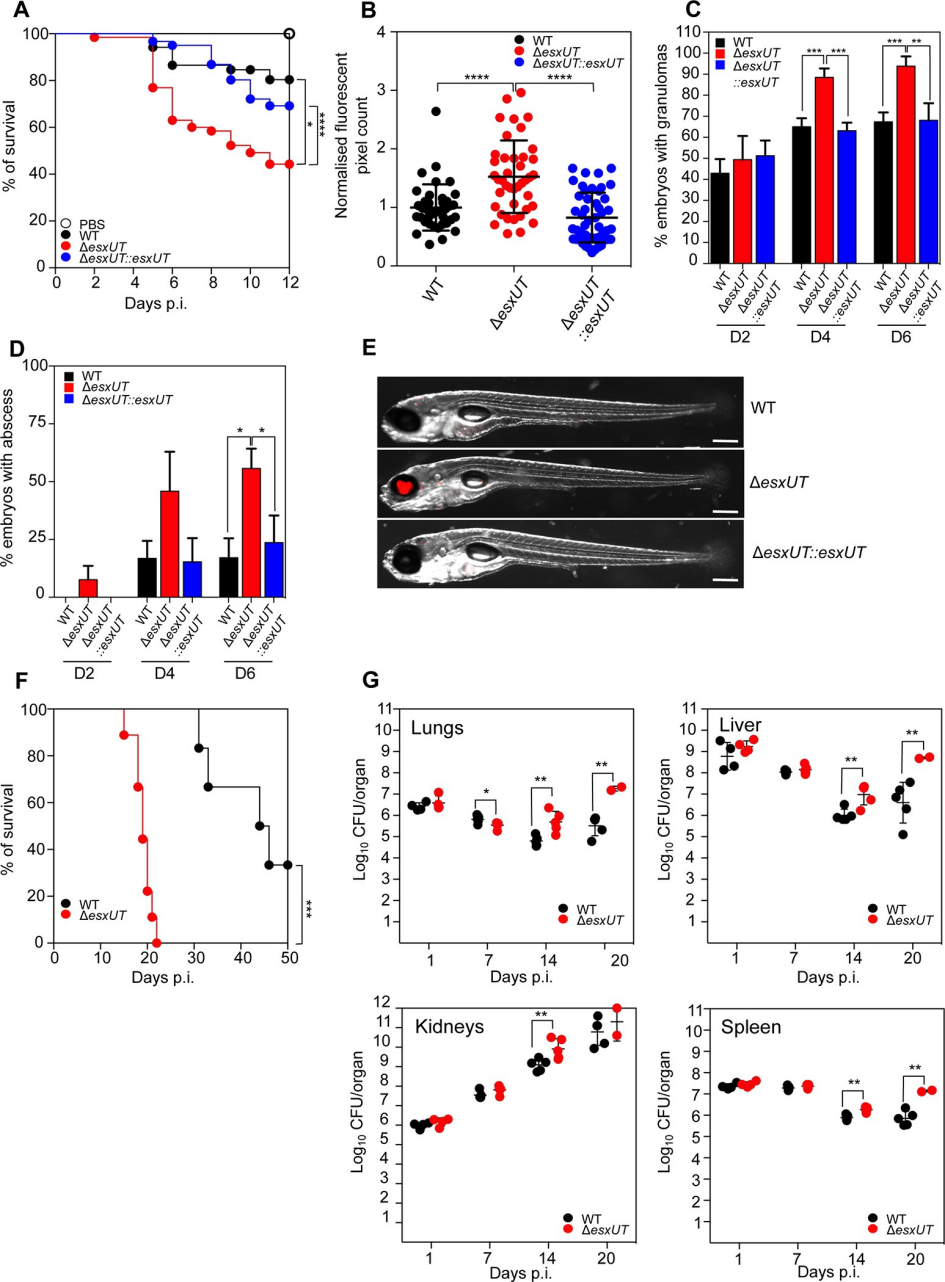
Δ*esxUT* is hypervirulent in zebrafish and C3HeB/FeJ mice. (A) Zebrafish were injected with 250–300 CFU of *M*. *abscessus* strains by tail vein injection and monitored daily for a 12-day infection period to determine relative embryo survival (B) At 6 dpi, zebrafish embryos were imaged under a fluorescent microscope and fluorescent pixel counts (FPC) calculated for each group using ImageJ. Each dot represents an individual embryo. (C) At 2, 4 and 6 dpi, infected zebrafish embryos were imaged using fluorescence microscopy to calculate the number of embryos with granulomas. Granulomas were identified on the basis of colocalisation of several fluorescent macrophages at an infection foci. (D) On days 2, 4, and 6 dpi, infected zebrafish embryos were imaged using fluorescence microscopy to calculate the proportion of embryos with abscesses. Abscesses were identified based on the relative size of infection foci far exceeding the surrounding recruited immune cells, representing uncontrolled bacterial growth. (E) Representative images of zebrafish infected with *M*. *abscessus* strains at day 6 post-infection. Red represents bacterial infection foci. Scale bar = 1mm. (F) Survival curves of C3HeB/FeJ mice after tail vein infection with 10^8^ CFU/mouse of WT (black line) and Δ*esxUT* (red line). (G) CFU counts of WT (black dots) and Δ*esxUT* (red dots) in lungs, liver, kidneys, and spleen at 1, 7, 14, and 20 dpi. Five mice were used per group and experiment was performed twice. *P* values were determined by ANOVA with Tukey’s test using GraphPad prism program (B, C and D) and unpaired *t* test (G); ns, not significant; *P < 0.05, **P < 0.01, ***P < 0.001, ****P < 0.0001. The log-rank (Mantel-Cox) test for Kaplan-Meier survival curves was used to assess survival statistics significance.

The previous results strongly suggest a physical interaction between the EsxU/EsxT complex and the membrane and the possibility that it allows *M*. *abscessus* to establish a phagosome-cytoplasm communication within the macrophage, which may in turn affect its pathogenicity. To explore the role of the EsxU/EsxT complex in the virulence of *M*. *abscessus* in the presence of a functional immune system, several previously established animal models were used [25,26]. In this context, the zebrafish (*Danio rerio*) model, which harbours only innate immunity (used as an acute infection model), and the C3HeB/FeJ mouse model, which possesses both innate and adaptive immunity (used as a persistent infection model), were exploited to address the contribution of EsxU/EsxT in *M*. *abscessus* pathogenesis. Survival curves in both animal models demonstrated that Δ*esxUT* confers a hypervirulent phenotype as compared with WT and complemented strains (Fig 4A and 4F). A significant increase in Δ*esxUT* bacterial burdens were found in zebrafish embryos (Fig 4B) as well as in C3HeB/FeJ mice (Fig 4G), presumably explaining the increased mortality observed in both animal models (Fig 4A and 4F). Furthermore, we observed a significantly higher proportion of embryos with granulomas at both 4 and 6 days post-infection (dpi), as well as a significantly increased number of embryos with abscesses at both 4 and 6 dpi in zebrafish infected with Δ*esxUT* as compared with WT and complemented strains (Fig 4C–4E). Similarly, we observed a significant increase in the number of CFU in the lungs, liver, kidneys and spleen at 14 and 20 dpi, confirming the increased growth of Δ*esxUT in vivo* (Fig 4G).

Overall, these findings support the view that deletion of *esxUT* in *M*. *abscessus* confers a hypervirulent phenotype in two complementary animal models.

## Discussion

Despite the increasing incidence of *M*. *abscessus* infections, little is known regarding the route of transmission of human-acquired infections [27]. Genetic evidence suggests that amoebae may represent an environmental reservoir, but this remains yet to be proven [7]. The amoebae/*M*. *abscessus* pairing unraveled important virulence determinants in *M*. *abscessus* [22,28], including a functional ESX-4 system [17]. This was subsequently confirmed in *M*. *tuberculosis* and *M*. *marinum*, by studying secretion of two ESX substrates, EsxE and EsxF [29] and of the CpnT toxin [8,30] and for which *M*. *tuberculosis* ESX-4 is essential for their secretion both *in vitro* and in macrophages [8]. However, *M*. *tuberculosis* and *M*. *marinum* both lack the essential EccE4 component of the ESX-4 secretory machinery, recently demonstrated to be essential for ESX-3 and ESX-5, which calls into question a functional and/or a stable ESX-4 secretory apparatus in these two species, yet this remains to be further investigated [1,2]. This is supported by a recent study indicating that ESX-4 from *M*. *marinum* does not secrete EsxT and EsxU [31]. It is, therefore, possible that the absence of *eccE4* in the *M*. *marinum esx-4* locus compromises the pore-forming capacity of ESX-4 and impaired the substrate transport activity. Indeed, *eccE4* is only present and conserved in the *M*. *abscessus-chelonae* complex (S4 Fig), possibly explaining the secretion of EsxU and EsxT observed in *M*. *abscessus*.

An important aspect of this work relies on the role of ESX-4 in phagosomal membrane permeabilization/disruption [8], a feature previously ascribed to ESX-1, but without incriminating the EsxU or EsxT secreted substrates, encoded by the *M*. *tuberculosis esx-4* locus. As mentioned above, mechanistic insights into the secretion of these molecules by ESX-4 in pathogenic mycobacteria are scarce, and mainly referring to *M*. *marinum* [31]. Using molecular and biophysical approaches, we confirmed that both EsxU and EsxT are substrates of *M*. *abscessus* ESX-4, forming a 1:1 heterodimer, which is secreted into the culture filtrate. Further biophysical experiments confirmed that the EsxU/EsxT complex is capable of interacting with artificial membranes and forming oligomeric arrangements permeabilizing the bilayer. A possible role in membrane permeabilization is also suggested by the macrophage experiments in which Δ*esxUT* remained inside the phagosome and was unable to induce phagosome membrane damage, as evidenced by the lack of a FRET signal in the phagosomal disruption FRET-signaling assay. As anticipated, phagosome-cytoplasm communication was restored in the complemented strain. Comparatively, Δ*eccB4* also failed to induce phagosomal membrane permeabilization [17]. The Δ*eccB4* secretome showed reduced secretion of EsxU and EsxT, confirming the association between EsxU/EsxT with phagosomal membrane permeabilization and the potential communication of *M*. *abscessus* with the macrophage cytosolic compartment [17]. Finally, immunofluorescence and immunoblotting analyses emphasized the localization of EsxU and EsxT in the cell wall periphery as well as in the culture filtrate. This localization suggests that EsxU and EsxT are ideally positioned to interact directly with the phagosomal membrane during the early intracellular stages of infection.

The phenotypic comparison between the two classes of mutants (Δ*eccB4 versus* Δ*esxUT*) revealed a major difference in their intracellular behavior. While Δ*esxUT* failed to show a defect in intracellular growth compared to the WT strain, presumably because it resides in phagosomes that do not acidify, this contrasts with the phagosomes containing Δ*eccB4* that acidify, leading to reduced intracellular survival [17]. The maintenance of *M*. *abscessus* within the phagosome, in contact with endocytosis and recycling pathways has been considered advantageous for intracellular multiplication of pathogenic mycobacteria [32]. It allows to escape apoptosis and autophagy, which are events encountered within the cytosol [33–35]. Furthermore, differences in phagosomal acidification support the existence of additional bacterial substrate(s), that may still be present in Δ*esxUT* but not in Δ*eccB4*. The absence of a functional secretion apparatus in Δ*eccB4* may impart the secretion of other compounds than just ESX-4 substrates. Indeed, deletion of *eccC4* in *M*. *marinum* resulted in elevated secretion of ESX-1 and ESX-5 protein substrates, underscoring the dialogue between ESX-4 and other T7SS [31]. In addition, dysfunctional ESX-4 may also impact the expression and/or biological activity of additional cell wall components, such as lipids. For instance, the synergistic role of phtiocerol dimycocerosates (PDIM) associated with ESX-1-secreted effectors has been demonstrated in *M*. *tuberculosis* [36,37]. In *M*. *abscessus*, deletion of *mmpL8*_*MAB*_ involved in the transport of another lipid, designated GDND, does not allow the bacteria to damage the phagosomal membrane [38]. These findings demonstrate the fine regulation and interaction between ESX-4 and its substrates in the pathophysiology of *M*. *abscessus* within phagocytic cells.

We show that Δ*esxUT* displays a hypervirulent phenotype in zebrafish and mice, supported by increased bacterial growth and mortality and enhanced granuloma and abscess formation, thus highlighting a central role for EsxU/EsxT in *M*. *abscessus* virulence. Given that EsxU/EsxT are largely found at the bacterial periphery and are capable of inducing phagosomal damage, one would predict that the absence of EsxU/EsxT leads to changes in the intracellular host-pathogen interface. This is not surprising, given that the smooth variant of *M*. *abscessus* used in this study is characterized by a tight phagosomal apposition surrounding the bacterial surface in macrophages [39]. Moreover, *M*. *abscessus* possesses a myriad of complex lipids on the outer surface, including glycopeptidolipids (GPL) and GDND, known to modulate infection outcomes and which are likely unaltered upon *esxUT* deletion. Interestingly, a hypervirulent phenotype was observed in adult zebrafish following infection with an ESX-5 mutant of *M*. *marinum* [40]. Similarly to Δ*esxUT*, an *esx-5* transposon mutant of *M*. *marinum* in EspG was accompanied by increased granuloma formation in adult zebrafish, while no growth defect was observed in THP-1 macrophages, as compared to the WT *M*. *marinum*. In addition, deletion of a gene encoding a PE-PGRS protein (a substrate of ESX-5 in *M*. *tuberculosis*) exhibits a hypervirulent phenotype in mice [41]. These analogies suggest that the ESX-4 substrates, EsxU and EsxT, can exhibit a virulence-associated phenotype similar to some ESX-5 substrates.

Unexpectedly, we did not observe specific antibody production against EsxU and EsxT during infection in either mouse or in human sera (S5 Fig). This result may be related to the early induction of *esxU* and *esxT* genes during macrophage infection, and a plausible explanation could rely on a partial or complete loss of EsxU/EsxT production in *M*. *abscessus* once it is established in the host, presumably as an adaptive response to increase its level of virulence. Future work will focus on describing the regulatory mechanisms governing *esxUT* expression under infection conditions. Overall, these observations lead to the tempting hypothesis linking the emergence of strict human and animal mycobacterial pathogens, such as *M*. *tuberculosis*, to the loss of ESX-4 substrate secretion resulting from the selection of non-functional ESX-4 systems lacking the essential EccE4 component.

## Materials and methods

### Ethics statement

Zebrafish experiments were completed under European Union guidelines for the handling of laboratory animals. Housing and husbandry were approved by the Direction Sanitaire et Vétérinaire de l’Hérault for the Montpellier Cell Biology Research Center zebrafish facility (Montpellier) (registration number C-34-175-39). Handling and experiments were approved by the Ministry of Higher Education and Research (APAFIS#24406–2020022815234677 V3). Mice experiments were performed according to institutional and national ethical guidelines (Agreement n°783223; approved by the Ministry of Higher Education and Research with APAFIS#11465-2016111417574906v4).

### Strains, cells, and culture conditions

*M*. *abscessus* CIP104536^T^ from the collection of Institut Pasteur, regrow from ATCC 19977^T^ [42] was grown in Middlebrook 7H9 broth (BD Difco) supplemented with 1% of glucose or 0.05% Tween 80 and 10% oleic acid, albumin, dextrose, catalase (OADC enrichment; BD Difco) at 37°C in the presence of antibiotics, when required. For bacterial selection, media were supplemented either with 25 mg/L of zeocin (Thermo Fisher Scientific) for Δ*esxUT*, 1 g/L hygromycin (Invivogen) for strains carrying pTEC27 (Addgene, plasmid 30182) and pMVH361 or with 250 mg/L kanamycin when harboring the pMV306 or pVV16 derivatives. J774 cells were maintained at 37°C in 5% CO_2_ using Dulbecco’s modified Eagle medium (DMEM) (Gibco) supplemented with 10% fetal bovine serum (FBS) (Gibco). J774 cells were allowed to adhere in a 24-well plate at a density of 5x10^4^ cells/well for 24 h at 37°C in 5% CO_2_.

### Identification of EsxT protein partners

Briefly, each bacterial pellet recovered from a 400 mL culture was resuspended in 2 mL of PBS containing 1 mM benzamidine. Subsequently, the bacteria were lysed using a bead beater and incubated in lysis buffer (50 mM Tris HCl pH 7.5, 250 mM NaCl, 5 mM EDTA, 1% NP40, 1% Triton, 1% Bridj96 and protease inhibitor [Benzamidine]). The supernatants were then subjected to immunoprecipitation following the instructions provided with the kit (GFP-Trap Agarose Beads from ChromoTek). Proteins bound to the beads were then separated and detected by silver staining. The remaining beads were subjected to trypsin treatment for mass spectrometry analysis.

### Mass spectrometry analysis

After denaturation at 100°C in 5% SDS, 5% β-mercaptoethanol, 1 mM EDTA, 10% glycerol, and 10 mM Tris pH 8 buffer for 3 min, protein samples were fractionated on a 10% acrylamide SDS-PAGE gel. The electrophoretic migration was stopped as soon as the protein sample entered 6 mm into the separating gel. The gel was briefly labeled with Coomassie Blue, and three bands, containing the whole sample, was cut. In gel, digestion of gel slices was performed as previously described [43]. An UltiMate 3000 RSLCnano System (Thermo Fisher Scientific) was used for separation of the protein digests. Peptides were processed and identified as previously described [44].

### Proteomic data analysis

MS/MS data were analyzed using search engine Mascot (version 2.4.0, Matrix Science, London, UK). Searches were performed with a tolerance on mass measurement of 10 ppm for precursor and 0.02 Da for-fragment ions, against a composite target-decoy database (181602*2 total entries) built with a *M*. *abscessus* Uniprot database (taxonomy 561007, march 2020, 4940 entries) fused with the sequences of EsxT-GFP. For each sample, peptides were filtered out according to the cut-off set for protein hits with one or more peptides taller than 9 residues, and a 1% false positive rate.

### EsxU and EsxT expression and purification in *M*. *smegmatis* and *E*. *coli*

*esxUT*, *esxU* and *esxT* were amplified from *M*. *abscessus* ATCC 19977 genomic DNA, cloned in the pMyNT vector using the SLiCE cloning method and transformed in *M*. *smegmatis* mc^2^155 *groEL1*ΔC cells. Gene expression was induced with 1% acetamide for 14 hrs at 37°C. Cell pellets were stored at −80°C until cells were lysed in a 50 mM Tris pH 8.5, 300 mM NaCl containing protease inhibitors and DNaseI using high-pressure emulsification. Unbroken cells were then removed by centrifugation (40 min at 12,000 *g*) and proteins were purified using 5 mL HiTrap TALON Crude (GE Healthcare) (5 CV equilibration, Load 100 mL sample, Wash 15 CV, 50 mM imidazole for 5 CV, 200 mM imidazole for 10 CV, 500 mM imidazole for 8 CV). The protein was then dialyzed into 50 mM Tris pH 8.5, 300 mM NaCl overnight in presence of TEV protease and ran through a 5 mL HiTrap TALON Crude (GE Healthcare). The resulting protein was then concentrated and run through an S75 preparative column (GE Healthcare). Concentration was performed using ultrafiltration (AMICON MWCO 3kDa). Protein purity was assessed using sodium dodecyl sulfate polyacrylamide gel electrophoresis.

For expression and purification in *E*. *coli*, *esxUT*, *esxU* and *esxT* were also cloned in the plasmid pET6×HN-C (Clontech, CA, USA), under the control of the T7/lac hybrid promoter, for expression in *E*. *coli* BL21(DE3) strain. Primers used for cloning are listed in S1 Table. Recombinant proteins were expressed after addition of IPTG at concentration from 0.1 to 10 mM in a 4 hr culture at 28°C. Proteins were purified either under native or denaturing conditions, according to manufacturer recommendations for Ni-NTA column (Qiagen). The presence of LPS was tested for recombinant proteins at the maximum concentration used for the assay in Fig 3 (80 μM) with the ToxinSensor kit, an endotoxin detection system from Genscript based on the LAL test. Proteins were tested after purification from *M*. *smegmatis* and also after passage through an endotoxin removal column (ToxinEraser, Genscript). Furthermore, the 7H9 culture medium was also tested. All values obtained for proteins and medium were out-of-range, i.e. below the lowest value of the standard curve (0.01 EU/mL).

### RNA extraction, qRT-PCR

RNA and cDNA were prepared from 5 mL of mid-logarithmic-phase bacterial cultures grown in 7H9. Control experiments without reverse transcriptase were performed for each RNA sample to rule out DNA contamination. The *sigA* gene was used as an internal control. The sequences of primers used for quantitative real-time PCR (qRT-PCR) are listed in S1 Table. For isolation of bacterial RNA from *M*. *abscessus*-infected J774.2 macrophages, RNA were extracted as previously described [28]. Each qRT-PCR was performed with three biological replicates.

### Δ*esxUT* mutant construction and complementation

Disruption of the *esxUT* gene marked with a zeocin resistance cassette was achieved using the recombineering system as described previously [22,45]. Briefly, the homology arms (1,000 bp upstream and downstream of *esxU* and *esxT*) were amplified from the genomic DNA of the CIP104536 (S) strain and the zeocin cassette was amplified from the pMVH361zeo. The amplicons were then cloned into the pUC19 plasmid by using Gibson technology (New England Biolabs). The Gibson product was electroporated into *E*. *coli*. The sequence including the homology arms and the zeocin resistance cassette called "allelic exchange sequence" (AES) was purified after cleavage by the restriction enzymes *PstI* and *KpnI*. The AES (300 ng) was electroporated into the wild-type strain containing the pJV53, which expresses the Che9c recombinase and a kanamycin resistance gene. Clones were selected on 7H11 supplemented with zeocin (50 mg/L) and kanamycin (250 mg/L) medium and screened by PCR to select those in which both recombination events had occurred. The target gene was then replaced by the zeocin resistance cassette and the pJV53 was removed from the strain. Complementation was performed after amplifying and cloning *esxUT* into the integrative pMVH361, as described previously [22].

Disruption of the *esxUT* gene unmarked in *M*. *abscessus* was performed as described previously [46]. Briefly, the pUX1-*katG* suicide vector was used to generate unmarked double deletion mutants in *M*. *abscessus* CIP104536^T^ (S). The left and right arms were amplified, restricted with *PacI/MfeI* and *MfeI/NheI*, respectively, and ligated to *PacI/NheI*-linearized pUX1-*katG*. Electrocompetent *M*. *abscessus* was transformed with pUX1-*katG*-*esxUT* to generate Δ*esxUT*. Selection of bacteria having undergone the first homologous recombination event was done by visual screening of red fluorescent colonies on 7H10^OADC^ supplemented with 250 μg/mL kanamycin. After subculturing the culture overnight in 7H9^T/OADC^ in the absence of kanamycin, bacterial suspensions were serially diluted and plated onto 7H10^OADC^ with 50 μg/mL INH to select for INH-resistant, Kan-sensitive, and non-fluorescent colonies. The DNA junctions were PCR sequenced to confirm the genotype of Δ*esxUT*.

Complementation was performed after amplifying and cloning *esxUT* in fusion with an HA-tagging sequence under the control of the *hsp60* promoter into the integrative pMV306, as described [46]. All primers used for the construction of the two mutants are listed in S1 Table.

### SEC-MALLS experiments

The EsxU/EsxT complex was purified as detailed above and dialyzed in both 20 mM Tris pH 8.0, 300 mM NaCl, 5% glycerol. EsxU/EsxT was injected at 10 mg/mL and experiments were performed at a flow rate of 0.8 mL/min. SEC-MALLS was performed using an Agilent 1260 Infinity II Bio-inert LC system and an S200 5/150 (GE Healthcare). Protein elution was detected by absorbance at 280 nm, and protein concentration was quantified with differential refractometry using an Optilab T-rEX detector (Wyatt). Light scattering data were measured with a miniDAWN TREOS multiangle light scattering detector (Wyatt). Molecular weights were computed from the concentration and light scattering data using the software ASTRA version 7.1.3.15 (Wyatt).

### Preparation of mycobacterial subcellular fractions and Western blotting

Briefly, 500 mL of mycobacterial cultures (wild-type smooth strain or *ΔesxUT* mutant) were grown to an OD_600_~0.3 in 7H9 medium containing 1% glucose at 37°C with shaking. Cells were collected by a 30-min centrifugation at 10,000 × *g* and the pellet was washed with PBS containing 1 mM benzamidine and resuspended in 2 mL PBS containing 1 mM benzamidine. Bacteria were lysed in a bead beater and the lysates transferred into 15 mL tubes and subjected to two rounds of sonication for 30 s on ice. Lysates were centrifuged twice at 4,000 × *g* for 10 min to remove unbroken cells. An aliquot of 200 μl of each supernatant (serving as total lysate sample) was stored at 4°C. The remaining supernatants were centrifuged at 16,000 × *g* for 30 min at 4°C to pellet cell walls. The supernatants were collected and ultra-centrifuged at 200,000 × *g* for 2 hrs at 4°C to separate plasma membranes (pellet) from the cytoplasmic fraction. The cell wall and plasma membrane fractions were each washed twice with PBS supplemented with 1 mM benzamidine and resuspended in 200 μl of PBS containing protease inhibitors. The cytoplasmic fractions were ultra-centrifuged one more time for 2 hrs at 200,000 × *g* to ensure that all residual membranes were removed. The protein concentration in each fraction was determined using the BSA kit and 50 μg of protein fraction was loaded on a gel for Western blotting using specific antibodies. Mouse anti-EsxU and EsxT sera were collected after DNA immunization (In-Cell-Art, Nantes, France), as previously described [47]. The specificity of the mouse serum (dilution, 1/500) was checked using a crude lysate from *E*. *coli* expressing the recombinant His-tagged EsxT or EsxU. A peroxidase-conjugated goat anti-mouse IgG (H+L, Southern Biotech) was then added (dilution, 1/5000) prior to detection using the peroxidase substrate. Mice anti-GroEL1 antibodies and anti-Ag85 antibodies were used at 1/5000 and 1/40 dilutions, respectively.

### Cell infection assays and flow cytometry analysis

Infection of J774.2 macrophages and *Acanthamoeba castellanii* infection were performed, as previously described [17]. After J774.2 infection in 12-well plates, CCF-4 FRET measurements were performed to study phagosomal rupture, as reported earlier [48].

### Phagosomal acidification assay

J774.2 cells were distributed into 24-well plates containing glass coverslips and were infected with *M*. *abscessus* expressing mCherry for 3 hrs at 37°C, 5% CO_2_. Infected cells were treated for 1 hr with amikacin (250 μg/mL) to kill extracellular bacteria and Green LysoTracker (dilution,1/2000) (Thermo Fisher Scientific) was added to each well. Colocalization of cell containing red fluorescent *M*. *abscessus* and LysoTracker green were counted under the microscope. The Pearson correlation was determined by analyzing more than 100 infected cells from at least 10 random fields with a Leica SP8-X confocal microscope. Pictures analyses and PCC evaluation were done with Icy and Image J software.

### Pore spanning lipid bilayer (NanoSpot assay)

Indium tin oxide (ITO)-coated glass slides were used for the electroformation of giant unilamellar vesicles (GUVs), made of dioleoyl-phosphatidylcholine (DOPC, Avanti Polar Lipids, Alabama, USA, Cat # 850375P) as described before [49,50]. For the preparation of lipid bilayers, a Si/SiO_2_ chip-based protocol based on the one described in [51], was previously developed and tested in the laboratory. The chips were prepared “in house” using silicon wafer with pre-cut and pre-etched chips (Micromotive GmbH, Mainz, Germany). 250 μl of SEC buffer (300 mM NaCl, 50 mM HEPES, pH 7.5) was added to each well and the chamber was sonicated for 2 min to remove air bubbles trapped in the cavities. 5 μL of Atto488 carboxy fluorescence dye (ATTO-TEC GmbH, North Rhine-Westphalia, Germany, Cat # AD 488–21) was added to each well (final concentration of 5 nM), followed by 50 μL of previously harvested GUVs. Chambers were centrifuged at 400 x g for 10 min at room temperature (Heraeus Megafuge 1.0, Germany) to allow rupture of GUVs and formation of lipid bilayers on top of the cavities. The chamber was placed in a dark camera kept at 37°C connected to a fluorescence inverted microscope (MORE integrated Life Cell Imaging System, Thermo Fisher Scientific FEI Munich formerly Till Photonics, Planegg, Germany) equipped with a 10×/0.45 objective (planapochromat 420640−9900−000, Zeiss, Jena, Germany), a FITC filterset (ET480_40x/ET535_50m/T510LPXRXT, Chroma, Rockingham, USA) and LED (transmitted light) and oligochrome (fluorescence) light sources (Thermo Fisher Scientific). 16-bit images were taken with a Andor clara low light imaging interline CCD camera (Andor Technology plc, Belfast, Northern Ireland) using Live Acquisition Software (LAS V. 2.2.2, Thermo Fisher Scientific). 20 μL of EsxU/EsxT was added to the well to initiate the experiment. Membrane permeabilization was detected in terms of fluorescence dye-efflux. In some experiments, 10 μL of 10 mg/mL TexasRed-labeled dextran molecules (Life technologies, Carlsbad, CA, USA) of 40 000 MW were added before the complex.

Image analysis (drift correction, identification of fluorescent areas, and extraction of time series) was done with ImageJ 1.52p (USA). Final data analysis and graphs were done with GraphPad Prism software version 9.0.

### Mice infection and bacterial load determination

C3HeB/FeJ mice were infected intravenously (i.v.) in the tail vein with 1 × 10^8^ CFU/mouse either with the wild-type or Δ*esxUT* strains [26]. Bacterial burden in the lungs, liver, spleen and kidneys was evaluated on day 1, 7, 14 and 20. Five mice per group were used, except on day 1 (four mice) and day 20 (two mice due to premature death). Differences between means were analyzed by two-way analysis of variance (ANOVA) and the Tukey’s post-test, allowing for multiple comparisons. n.s., non-significant; *, P<0.05; **, P< 0.01; ***, P< 0.001. Experiment was performed twice.

### Zebrafish experiments

Zebrafish infections were completed as previously described [25,52]. The golden mutant zebrafish line and the Tg(*mpeg*:*mCherry*) red fluorescent macrophage line were used as previously described. Embryos at 30 hrs post-fertilisation were dechorionated and anaesthetised in tricaine prior to caudal vein injection with 2–3 nL of *M*. *abscessus* strains (~100 CFU/nL) expressing Tdtomato fluorescent protein under the control of pTEC27 plasmid. After infection, embryos were recovered in fresh embryo water and placed into 24-well plates with 2 embryos/well. Embryos were monitored daily, with dead embryos removed from the well daily until 12 days post-infection. At 2, 4 and 6 days post-infection, embryos were anaesthetised in tricaine and placed on 3% methylcellulose (w/v) for fluorescent microscopy using Zeiss Axio Zoom.V16 coupled with an Axiocam 503 mono (Zeiss). Granulomas were quantified based on the bacterial co-localisation with macrophage fluorescent signal. Bacterial burden was quantified in ImageJ using the ‘Analyze particles’ function to quantify bacterial fluorescence. Abscesses were quantified based on the large uncontrolled extracellular growth of fluorescent bacteria that exceeded 100 μm in diameter. All experiments were performed in at least three independent experiments.

### EsxU/EsxT ELISA

A total of 173 sera from CF patients, including 33 patients with *M*. *abscessus* positive cultures, and 31 non-CF healthy controls were analyzed. The distribution of the different groups depending on the results of sputum cultures were previously described against the PLC protein [53]. IgG levels were evaluated by indirect ELISA assays using a EsxU/EsxT recombinant protein complex or the PLC protein with sera diluted at 1/400. ELISA assays were developed as previously described [53] with alkaline phosphatase-conjugated goat anti-human IgG (Southern biotechnology, Birmingham, USA) diluted in PBS containing 0.05% (v/v) Tween 20 (PBS-T) and 0.5% BSA (PBS-T-BSA). OD mean ± standard deviation was calculated for each patient group.

## Supporting information

S1 Fig Confirmation of EsxU and EsxT partnership by GFP-pull-down experiments and mass spectrometry. (A) EsxU and EsxT were fused to a C-terminal GFP tag under the control of the *hsp60* promoter. Molecular weight in kDa is indicated at the bottom of each schema. (B) Western blot analysis of Δ*esxUT* strain expressing GFP, EsxT-GFP, EsxU-GFP or EsxUT-GFP fusion proteins using anti-GFP antibodies. (C) Illustration of the affinibody GFP nanobody pull-down kit. (D) Silver-stained gel showing that a major band migrating between 10 and 15 kDa, presumably corresponding to EsxU, was detected after immunoprecipitation using anti-GFP nanobodies. The bacterial strain stably expressing GFP was used as a negative control. The eluates of GFP and EsxT-GFP beads were subjected to mass spectrometry analysis. (E) Selection of the most representative proteins belonging to the EsxT interaction network.
(TIF)

S2 Fig Construction of Δ*esxUT* knockout mutant. (A Left) Strategy for generating Δ*esxUT* by homologous recombination between the allelic exchange sequence and the genomic DNA of *M*. *abscessus*. The *esxUT* gene is replaced with a zeocin resistance cassette. The arrows represent the primers used to verify the mutation. *(Right)* Primers in *esxU* and *esxT* were used as controls. (B) Comparison of WT (black), Δ*esxUT* (red) and Δ*esxUT*::*esxUT* (blue) bacterial growth in 7H9 tween medium. (C) Colony morphology of WT, Δ*esxUT* and Δ*esxUT*::*esxUT* strains under the binocular microscope. (D) Relative intracellular survival of WT (black), Δ*esxUT* (red), and Δ*esxUT*::*esxUT* (blue) strains as determined by CFU counts during co-culture with the amoeba *Acanthamoeba castellanii*. Data are representative of three independent experiments and represent means ± SEM. *P* values were determined by ANOVA with Tukey’s test using the GraphPad prism program. ns, not-significant.
(TIF)

S3 Fig The unmarked *ΔesxUT* behaves similarly to the recombineering *ΔesxUT* mutant. (A) The *esx4UT* genes are sandwiched between *MAB_3755c* and the *50S ribosomal protein L13* gene. The plasmid pUX1-katG-*esxUT* was generated to remove the *esxUT* genes by double homologous recombination. Black arrows represent the primers used for PCR analysis. (B) PCR analysis demonstrating deletion of *esxUT* genes. Genomic DNA from WT bacteria was used to amplify the intact *esxUT* locus. The mutant amplicon (2127 bp) was subjected to sequencing to confirm the proper deletion of *esxUT*. (C) The Δ*esxUT* mutant was complemented using the integrative pMV306 carrying either *esxU*, *esxT* or *esxUT* fused to a C-terminal HA tag under the control of the *hsp60* promoter. The molecular weight in kDa is indicated at the bottom of each schema. (D) Western blot analysis of complemented strains expressing single or combined subunits fused to an HA tag using anti-HA and anti-KasA (loading control) antibodies. (E) Relative intracellular survival of WT (black), Δ*esxUT* (red), and Δ*esxUT*::*esxUT* (blue) strains as determined by CFU counts during infection in THP-1 human macrophages (MΦ) at an MOI of 10:1. (F) Phagosomal rupture detected by CCF-4 FRET-based flow cytometry. Results are depicted as signal overlays per group with 1,000,000 events per condition acquired in WT (dark line), heat-killed (dark dotted line), Δ*esxUT* (red line), and uninfected (NI, light gray filled curve) strains. Data are representative of two independent experiments.
(TIF)

S4 Fig Comparison of *M*. *abscessus* and *M*. *chelonae* ESX-4. *esx-4* locus composition of *M*. *tuberculosis*, *M*. *marinum*, *M*. *avium*, *M*. *abscessus*, *M*. *smegmatis* and *M*. *chelonae* composed by esx genes (red arrows), unknown gene (dark blue), *eccC* (blue), *eccD* (orange), *mycP* (cyan), *eccB* (green), *eccE* (purple). Schematic designed on gene graphics. (B) Protein alignment of *M*. *abscessus* and *M*. *chelonae* EsxU, (C) EsxT and (D) EccE. Difference of blue reflects protein identity. Alignments were performed on ClustalW2 and visualized on Jalview.
(TIF)

S5 Fig Lack of immunological response against EsxU/EsxT during infection. (A) Five BALB/c mice per group were infected sub-cutaneously twice (with a four weeks latency/interval) with 10^6^ CFU WT (orange line), Δ*esxUT* (yellow line) or Δ*esxUT*::*esxUT* (pink line). Antibody response against EsxU/EsxT was determined by ELISA two weeks after the second infection. Sera from uninfected mice (green line) were also analyzed to assess the baseline of a specific antibody binding. Superposed is the antibody response against Phospholipase C (PLC) (gray line). (B) Seric anti-EsxUT IgG response in different groups of CF patients infected with various pathogens: *Mabs*, MAC, other NTM, Pa, NTM^-^/Pa^-^, F and in healthy controls (HC) using purified recombinant EsxU/EsxT as antigen. Each dot represents one patient in the scatterplots. CF patients were classified based on their culture-positivity: for *M*. *abscessus* (*Mabs*), mycobacteria of the *M*. *avium* complex (*M*. *avium*, *M*. *chimaera or M*. *intracellulare)*, for other non-tuberculous mycobacteria (other NTM), for *Pseudomonas aeruginosa* (Pa), for fungi (F). NTM/Pa represents CF patients without a positive culture for NTM and *Pseudomonas aeruginosa*. Finally, the HC group represents healthy subjects who are not CF patients. (C) Mean IgG responses (± SD) against *M*. *abscessus* PLC in each group, as previously reported [53]. *P* values were determined by unpaired *t* test; *P < 0.05, **P < 0.01, ***P < 0.001, ****P < 0.0001.
(TIF)

S1 Table Primers used in this study. (DOCX)
