# Supplementary figures and images for: The ESX-4 substrates, EsxU and EsxT, modulate Mycobacterium abscessus fitness
Source: PLoS Pathog. 2022 Aug 12;18(8):e1010771. doi: 10.1371/journal.ppat.1010771 (PMC9401124; doi:10.1371/journal.ppat.1010771)

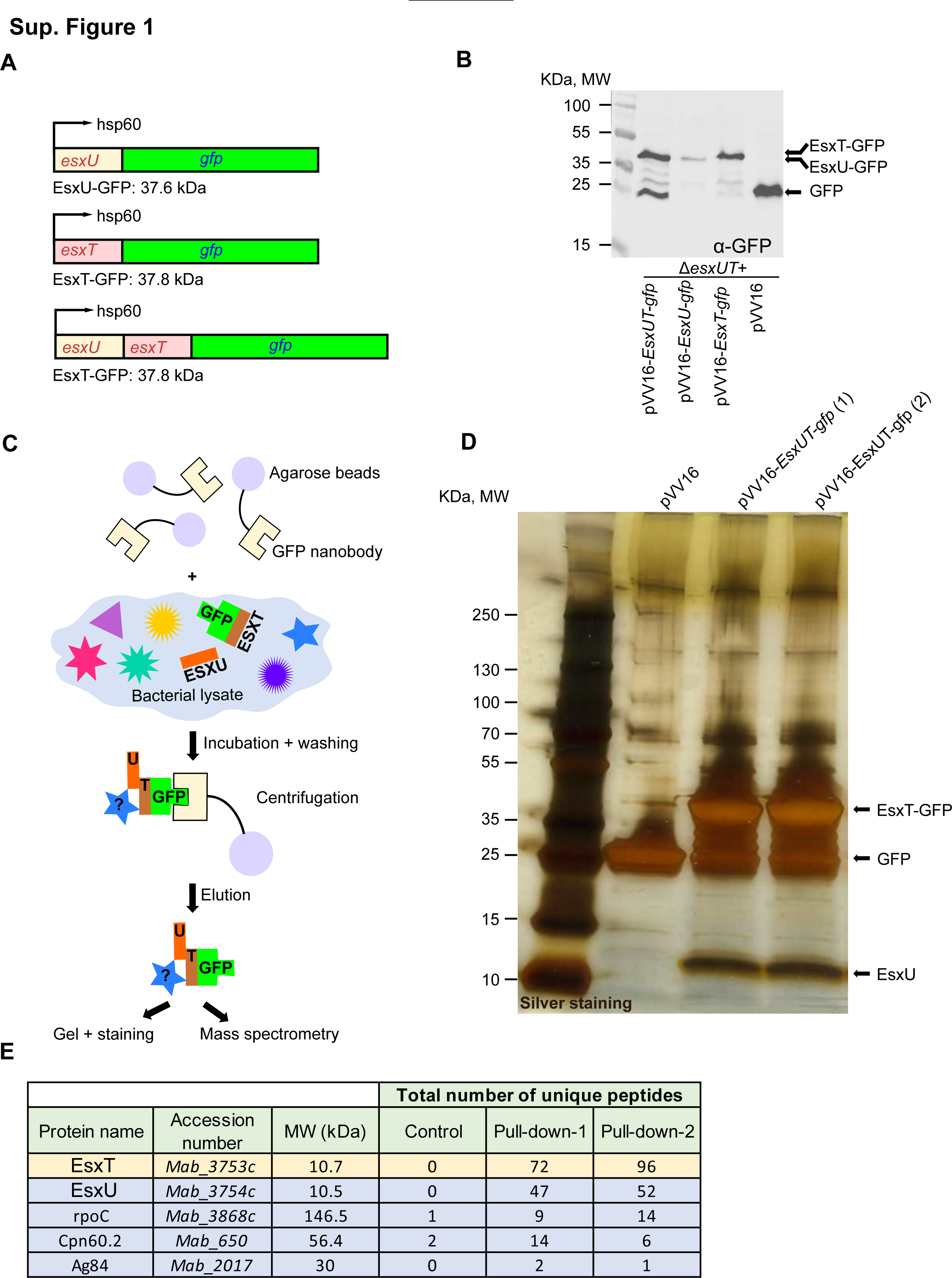

Supplement: S1 Fig — (A) EsxU and EsxT were fused to a C-terminal GFP tag under the control of the hsp60 promoter. Molecular weight in kDa is indicated at the bottom of each schema. (B) Western blot analysis of ΔesxUT strain expressing GFP, EsxT-GFP, EsxU-GFP or EsxUT-GFP fusion proteins using anti-GFP antibodies. (C) Illustration of the affinibody GFP nanobody pull-down kit. (D) Silver-stained gel showing that a major band migrating between 10 and 15 kDa, presumably corresponding to EsxU, was detected after immunoprecipitation using anti-GFP nanobodies. The bacterial strain stably expressing GFP was used as a negative control. The eluates of GFP and EsxT-GFP beads were subjected to mass spectrometry analysis. (E) Selection of the most representative proteins belonging to the EsxT interaction network. (TIF) [file ppat.1010771.s001.tif]

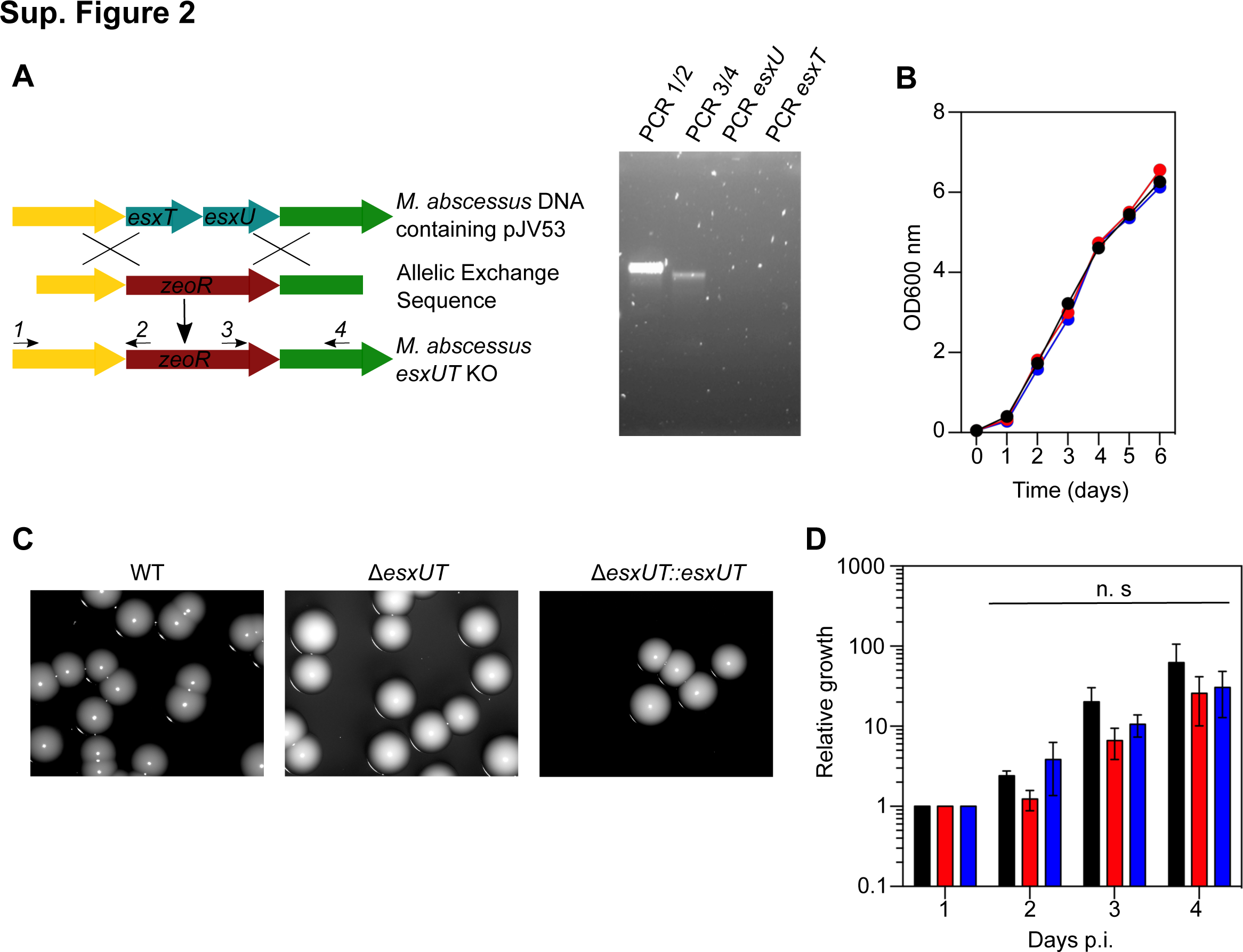

Supplement: S2 Fig — (A Left) Strategy for generating ΔesxUT by homologous recombination between the allelic exchange sequence and the genomic DNA of M. abscessus. The esxUT gene is replaced with a zeocin resistance cassette. The arrows represent the primers used to verify the mutation. (Right) Primers in esxU and esxT were used as controls. (B) Comparison of WT (black), ΔesxUT (red) and ΔesxUT::esxUT (blue) bacterial growth in 7H9 tween medium. (C) Colony morphology of WT, ΔesxUT and ΔesxUT::esxUT strains under the binocular microscope. (D) Relative intracellular survival of WT (black), ΔesxUT (red), and ΔesxUT::esxUT (blue) strains as determined by CFU counts during co-culture with the amoeba Acanthamoeba castellanii. Data are representative of three independent experiments and represent means ± SEM. P values were determined by ANOVA with Tukey’s test using the GraphPad prism program. ns, not-significant. (TIF) [file ppat.1010771.s002.tif]

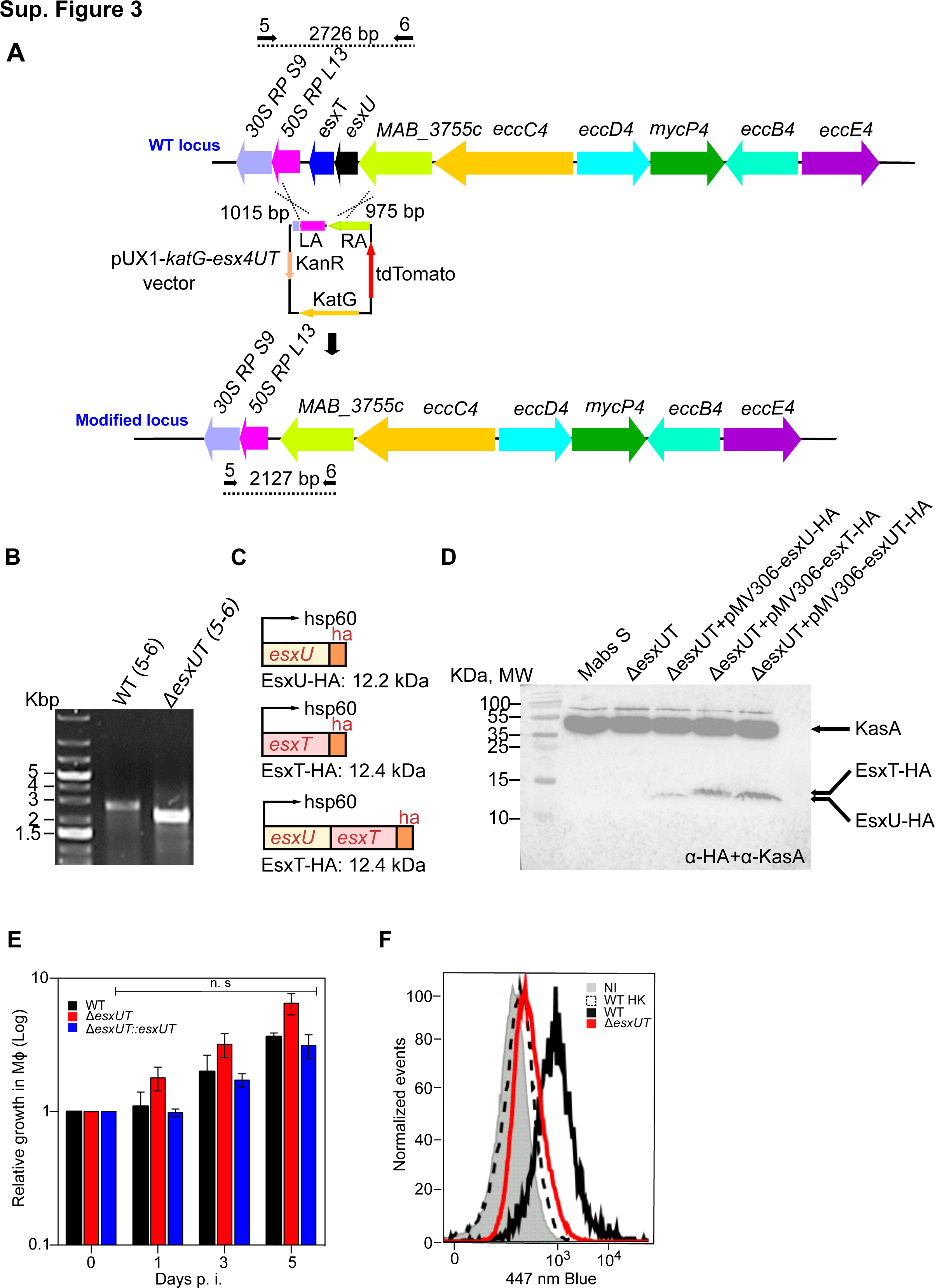

Supplement: S3 Fig — (A) The esx4UT genes are sandwiched between MAB_3755c and the 50S ribosomal protein L13 gene. The plasmid pUX1-katG-esxUT was generated to remove the esxUT genes by double homologous recombination. Black arrows represent the primers used for PCR analysis. (B) PCR analysis demonstrating deletion of esxUT genes. Genomic DNA from WT bacteria was used to amplify the intact esxUT locus. The mutant amplicon (2127 bp) was subjected to sequencing to confirm the proper deletion of esxUT. (C) The ΔesxUT mutant was complemented using the integrative pMV306 carrying either esxU, esxT or esxUT fused to a C-terminal HA tag under the control of the hsp60 promoter. The molecular weight in kDa is indicated at the bottom of each schema. (D) Western blot analysis of complemented strains expressing single or combined subunits fused to an HA tag using anti-HA and anti-KasA (loading control) antibodies. (E) Relative intracellular survival of WT (black), ΔesxUT (red), and ΔesxUT::esxUT (blue) strains as determined by CFU counts during infection in THP-1 human macrophages (MΦ) at an MOI of 10:1. (F) Phagosomal rupture detected by CCF-4 FRET-based flow cytometry. Results are depicted as signal overlays per group with 1,000,000 events per condition acquired in WT (dark line), heat-killed (dark dotted line), ΔesxUT (red line), and uninfected (NI, light gray filled curve) strains. Data are representative of two independent experiments. (TIF) [file ppat.1010771.s003.tif]

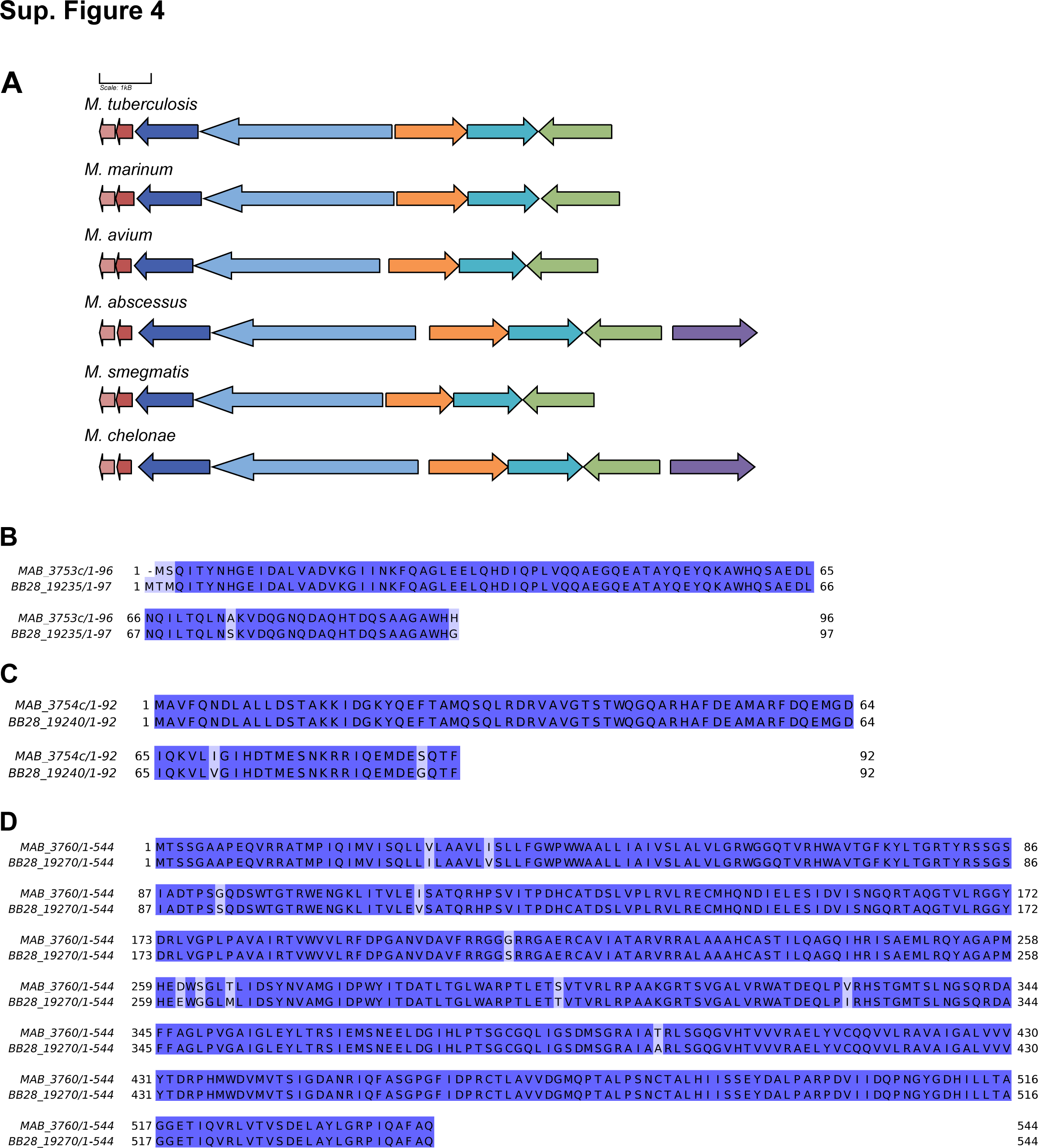

Supplement: S4 Fig — esx-4 locus composition of M. tuberculosis, M. marinum, M. avium, M. abscessus, M. smegmatis and M. chelonae composed by esx genes (red arrows), unknown gene (dark blue), eccC (blue), eccD (orange), mycP (cyan), eccB (green), eccE (purple). Schematic designed on gene graphics. (B) Protein alignment of M. abscessus and M. chelonae EsxU, (C) EsxT and (D) EccE. Difference of blue reflects protein identity. Alignments were performed on ClustalW2 and visualized on Jalview. (TIF) [file ppat.1010771.s004.tif]

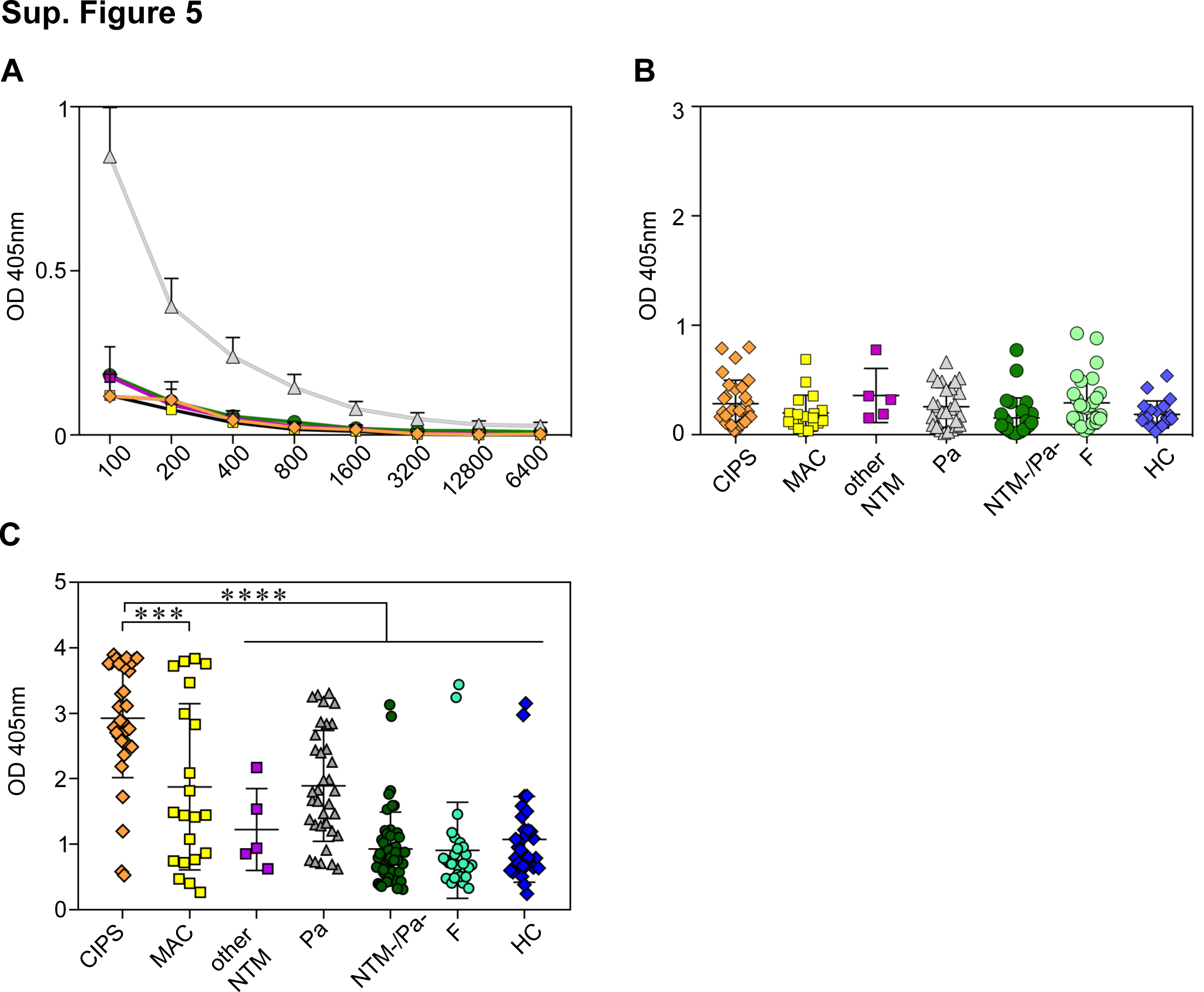

Supplement: S5 Fig — (A) Five BALB/c mice per group were infected sub-cutaneously twice (with a four weeks latency/interval) with 106 CFU WT (orange line), ΔesxUT (yellow line) or ΔesxUT::esxUT (pink line). Antibody response against EsxU/EsxT was determined by ELISA two weeks after the second infection. Sera from uninfected mice (green line) were also analyzed to assess the baseline of a specific antibody binding. Superposed is the antibody response against Phospholipase C (PLC) (gray line). (B) Seric anti-EsxUT IgG response in different groups of CF patients infected with various pathogens: Mabs, MAC, other NTM, Pa, NTM-/Pa-, F and in healthy controls (HC) using purified recombinant EsxU/EsxT as antigen. Each dot represents one patient in the scatterplots. CF patients were classified based on their culture-positivity: for M. abscessus (Mabs), mycobacteria of the M. avium complex (M. avium, M. chimaera or M. intracellulare), for other non-tuberculous mycobacteria (other NTM), for Pseudomonas aeruginosa (Pa), for fungi (F). NTM/Pa represents CF patients without a positive culture for NTM and Pseudomonas aeruginosa. Finally, the HC group represents healthy subjects who are not CF patients. (C) Mean IgG responses (± SD) against M. abscessus PLC in each group, as previously reported [53]. P values were determined by unpaired t test; *P < 0.05, **P < 0.01, ***P < 0.001, ****P < 0.0001. (TIF) [file ppat.1010771.s005.tif]
