# Supplementary material for: The ESX-4 substrates, EsxU and EsxT, modulate Mycobacterium abscessus fitness
Source: PLoS Pathog. 2022 Aug 12;18(8):e1010771. doi: 10.1371/journal.ppat.1010771 (PMC9401124; doi:10.1371/journal.ppat.1010771)
Supplement: S1 Table — (DOCX) [file ppat.1010771.s006.docx]

S1 Table. Primers used in this study

| **Number** | **5’→3’ sequence** | **Restriction**  **site** | **F (sense),**  **R (antisense)** |
| --- | --- | --- | --- |
| **Cloning in pUX1-*katG*** | | | |
| 1 | gaga**TTAATTAA**tagtccgcgatgaa  cgtccaggagt | PacI | To clone the right arm of  *esxUT* in pUX1-*katG* (F) |
| 2 | gaga**CAATTG**ATTCTGAAAAACAGCC  ACttcagttcctccc | MfeI | To clone the right arm of  *esxUT* in pUX1-*katG* (R) |
| 3 | gaga**CAATTG**cactgacccttccggtcaatctgtag | MfeI | To clone the left arm of  *esxUT* in pUX1-*katG* (F) |
| 4 | gaga**GCTAGC**ttgatgagctgctggtgcaccttgttc | NheI | To clone the left arm of  *esxUT* in pUX1-*katG* (R) |
| **Primers to verify double homologous recombination** | | | |
| 5 | ttcgccaccttgaggatcgatcctg | - | To check double homologous recombination Δ*esxUT* (F) |
| 6 | tcaggtgggcgtaaatgtccacgct | - | To check double homologous recombination Δ*esxUT* (R) |
| **Primers to sequence modified *esxUT* locus** | | | |
| 7 | catggagaaggagcctcaa | - | To sequence Δ*esxUT* locus  (F) |
| 8 | attgatgatgatgacgaaatcgc | - | To sequence Δ*esxUT* locus  (R) |
| **Cloning in pMV306-KanR** | | | |
| 9 | gaga**CAATTG**CCGTGGCTGTTTTTCAGAATGACCTGGC | MfeI | To clone the *esxU* ORF  in pMV306 (F) |
| 10 | gaga**AAGCTT**CTAAGCGTAATCTGGAACATCGTATGGGTAG  AAGGTCTGGCTCTCGTCCATC | HindIII | To clone the *esxU* ORF  in pMV306 (R) |
| 11 | gaga**CAATTG**CCATGAGCCAGATTACTTACAACCACGGC | MfeI | To clone the *esxT* ORF  in pMV306 (F) |
| 12 | gaga**AAGCTT**CTAAGCGTAATCTGGAACATCGTATGG  GTAGTGGTGCCAGGCGCCGG | HindIII | To clone the *esxT* ORF  in pMV306 (R) |
| 13 | gaga**CAATTG**CCGTGGCTGTTTTTCAGAATGACCTGGC | MfeI | To clone the *esxUT* ORFs  in pMV306 (F) |
| 14 | gaga**AAGCTT**CTAAGCGTAATCTGGAACATCGTATGG  GTAGTGGTGCCAGGCGCCGG | HindIII | To clone the *esx4UT* ORFs  in pMV306 (R) |
| **Cloning in pVV16-KanR** | | | |
| 15 | gaga**CATATG**GTGGCTGTTTTTCAGAATGACCTGGC | NdeI | To clone the *esxU* ORF  in pVV16 (F) |
| 16 | gaga**GGATCC**GAAGGTCTGGCTCTCGTCCATC | BamHI | To clone the *esxU* ORF  in pVV16 (R) |
| 17 | gaga**CATATG**ATGAGCCAGATTACTTACAACCACGGC | NdeI | To clone the *esxT* ORF  in pVV16 (F) |
| 18 | gaga**GGATCC**GTGGTGCCAGGCGCCG | BamHI | To clone the *esxT* ORF  in pVV16 (R) |
| 19 | gaga**CATATG**GTGGCTGTTTTTCAGAATGACCTGGC | NdeI | To clone the *esxUT* ORFs  in pVV16 (F) |
| 20 | gaga**GGATCC**GTGGTGCCAGGCGCCG | BamHI | To clone the *esxUT* ORFs  in pVV16 (R) |
| **Cloning in pUC19 zeo** | | | |
| 21 | gccaagcttgcatgcctgcagATCGATCCTGGGAGACCAG |  | Gibson_EsxU_Left_Fwd |
| 22 | gttgctctagAAAACAGCCACTTCAGTTCC |  | Gibson_EsxU_Left |
| 23 | tggctgttttCTAGAGCAACGAAAGGCTCAG |  | Gibson_Zeocine_Fwd |
| 24 | gtcagtggtgGCGGAACGCTCAGTGGAAC |  | Gibson_Zeocine_Rev |
| 25 | agcgttccgcCACCACTGACCCTTCCGG |  | Gibson_EsxT_Right_Fwd |
| 26 | agtgaattcgagctcggtaccGATGAGCTGCTGGTGCAC |  | Gibson_EsxT_Right_Rev |
| 27 | agtgaattcgagctcggtaccGATGAGCTGCTGGTGCAC |  | EsxUT_pMVH361_Fwd |
| 28 | taactacgtcgacatcgataTCAGTGGTGCCAGGCGC |  | EsxUT_pMVH361_Rev |
| **Primer to check if the *esxUT* gene are replaced by zeocine** | | | |
| 29 | GTCCGACGGTGATGAACCCGCTGCG |  | To check double homologous recombination Δ*esxUT* primer1 |
| 30 | GAGCACCGGAACGGCACTGGTCAAC |  | To check double homologous recombination Δ*esxUT* primer 2 |
| 31 | GCATGACCAAAATCCCTTAACGTGAGTTTTCG |  | To check double homologous recombination Δ*esxUT* primer 3 |
| 32 | GTGGGCGTAAATGTCCACGCTGTC |  | To check double homologous recombination Δ*esxUT* primer 4 |
| **Cloning in pET6HN** | | | |
| 33 | ATATCCAAGCTTGTGGCTGTTTTTCAGAATGACCT- | HindIII | pET6HN-MAB_3754-HindIII |
| 34 | CCGGAATTCGCGAAGGTCTGGCTCTCGTCC | EcoRI | pET6HN-MAB_3754-EcoRI |
| 35 | ATATCCAAGCTTATGAGCCAGATTACTTACAACCAC | HindIII | pET6HN-MAB_3753c-HindIII |
| 36 | CCGGAATTCGCGTGGTGCCAGGCGCCG | EcoRI | pET6HN-MAB_3753c-EcoRI |
| **qPCR primers** | | | |
| 37 | TACCAGGAGTTCACGGCGAT |  | qRT_MAB_3754_Fwd |
| 38 | CATCTCCTGGTCGAAGCGAG |  | qRT_MAB_3754_Rev |
| 39 | CGCTGGTTGCTGATGTCAAG |  | qRT_MAB_3753_Fwd |
| 40 | GTACTCCTGGTACGCAGTGG |  | qRT_MAB_3753_Rev |
| 41 | TCCGAGAAAGACAAGGCTTC |  | SigA_Fwd |
| 42 | CCAGCTCAACTTCCTCTTCG |  | SigA_Rev |
